# Editorial: Genetic regulatory mechanisms of osmotic stress response in plants

**DOI:** 10.3389/fpls.2025.1555255

**Published:** 2025-01-28

**Authors:** Hye-Yeon Seok, Byeong-ha Lee, Yong-Hwan Moon

**Affiliations:** ^1^ Institute of Systems Biology, Pusan National University, Busan, Republic of Korea; ^2^ Department of Life Science, Sogang University, Seoul, Republic of Korea; ^3^ Department of Integrated Biological Science, Pusan National University, Busan, Republic of Korea; ^4^ Department of Molecular Biology, Pusan National University, Busan, Republic of Korea

**Keywords:** water-deficit stress, drought stress, abiotic stress, heat stress, stress response, stress tolerance

Plant adaptation to osmotic stress—a consequence of drought, salinity, and other abiotic stresses—is a critical focus in plant biology, given its implications for agricultural productivity and food security (Lim et al., 2015; Zareen et al., 2024). In the signal transduction network, from the perception of stress signals to stress-responsive gene expression, various transcription factors and *cis*-regulatory elements in stress-responsive promoters play pivotal roles in plant adaptation to abiotic stresses. Additionally, post-transcriptional regulation of gene expression is mediated by RNA metabolism (Lee et al., 2006; Kim et al., 2017; Park et al., 2024). The balance between transcriptional activators and repressors is vital for proper gene expression and responses to abiotic stresses (Seok et al., 2022). This Research Topic consolidates recent advancements in understanding the genetic regulatory mechanisms underlying osmotic stress responses, featuring seven studies exploring plant adaptation’s molecular, biochemical, and genomic dimensions.

## Insights into NAC transcription factors

Li et al. examined the role of the NAC transcription factor MdNAC29 in apples, revealing its negative regulation of drought tolerance. Overexpression of *MdNAC29* resulted in increased oxidative damage, reduced chlorophyll content, and downregulation of drought-responsive genes like *MdDREB2A*. The interaction between MdNAC29 and F-box protein MdPP2-B10 further highlighted its regulatory role in transcriptional repression under drought conditions. This study underscores the complexity of transcription factor-mediated gene expression and provides a basis for improving drought tolerance through genetic engineering.

## Catalase gene function in osmotic stress

Xu et al. identified a novel catalase gene, *PtCAT2*, from *Pinellia ternata*, which enhances drought tolerance in *Arabidopsis thaliana*. Overexpression of *PtCAT2* increased catalase activity by five-fold, leading to improveed reactive oxygen species (ROS) scavenging and a reduction in oxidative damage. This study highlights the pivotal role of ROS balance in osmotic stress responses and positions *PtCAT2* as a candidate for genetic interventions in drought-sensitive crops.

## Proteomic approaches to drought stress

Gao et al. employed proteomic analysis to elucidate the drought stress responses in leaves and roots of foxtail millet (*Setaria italica*). The study identified significant differences, with leaves primarily altering photosynthesis-related proteins and roots modifying a greater number of proteins involved in metabolites metabolism and stress-defense during both drought and recovery phases. These findings underscore the importance of tissue-specific drought adaptations for the development of drought-stress tolerant crops.

## FRF gene family in barley

He et al. investigated the functions of *HvFRF9* because it showed high expression in vascular tissues and root epidermis and was strongly induced by drought stress after a genome-wide analysis of the FAR-RED ELONGATED HYPOCOTYL3-RELATED FACTOR (FRF) gene family in barley (*Hordeum vulgare*). *HvFRF9*-overexpressing *Arabidopsis* demonstrated reduced osmotic stress and increased antioxidant ability by enhancing proline content and antioxidant enzyme activities. This study provides valuable insights into barley’s genetic adaptability and offers promising genetic targets for crop improvement.

## Root adaptation to osmotic gradients

Piríz-Pezzutto et al. developed an innovative *in vitro* osmotic gradient system to study *Arabidopsis* root adaptation in natural field conditions, where roots encounter increasing osmotic potential while exploring the soil. This system revealed that roots grown under osmotic gradients sustained higher growth rates and exhibited distinct changes in the expression of certain genes compared to those subjected to uniform osmotic shock. Findings from this study emphasize the importance of mimicking field conditions to uncover mechanisms of adaptive root growth.

## Role of MdKAI2 in apple osmotic stress resistance

Guo et al. explored the function of MdKAI2, a receptor for karrikins (KARs), in regulating osmotic stress resistance in apples. The study demonstrated that MdKAI2 positively regulates stress tolerance by enhancing ROS scavenging, promoting flavonoid biosynthesis, and increasing osmoregulatory substances. RNA-sequencing analysis of MdKAI2-overexpressing apple calli revealed that MdKAI2 modulates the expression of various transcription factors and genes involved in the MAPK signaling pathway. These findings open avenues for leveraging KAR-related pathways to improve tree crop resilience.

## Influence of ploidy on stress tolerance in citrus

To investigate the influence of ploidy levels on salt stress tolerance in citrus, Bonnin et al. conducted a comparative transcriptomic analysis of diploid and tetraploid citrus genotypes under salt stress. Tetraploid genotypes exhibited enhanced oxidative stress tolerance and differential expression of genes involved in cell wall remodeling, sugar metabolism, and antioxidant responses. This research highlights the potential of polyploidy in improving abiotic stress resilience in perennial fruit crops.

## Future directions

These studies collectively advance our understanding of osmotic stress response mechanisms across diverse plant systems. They highlight the importance of transcription factors, enzymatic regulators, proteomic changes, and genomic adaptations in mitigating osmotic stress. Future research should prioritize integrative multi-omics approaches, field-like experimental designs, and the translation of findings into crop improvement programs (Seok et al., 2023).

The contributions in this Research Topic shed light on the plant resilience strategies and provide practical insights for developing stress-tolerant crops essential for sustainable agriculture in an era of climate unpredictability by dissecting the molecular and genetic pathways of osmotic stress tolerance.
